# A Clinical Report of Nonsyndromic Concomitant Hypo-Hyperdontia

**DOI:** 10.1155/2013/598727

**Published:** 2013-09-28

**Authors:** Siddarth Gupta, Hashmat Popat

**Affiliations:** ^1^School of Dentistry, Cardiff University, Heath Park, Cardiff CF14 4XY, UK; ^2^Applied Clinical Research and Public Health, School of Dentistry, Cardiff University, Heath Park, Cardiff CF14 4XY, UK

## Abstract

Although hypodontia and supernumerary teeth are often considered as mutually exclusive conditions, this case report presents an unusual case of hypodontia and a supernumerary tooth occurring simultaneously. An adolescent male was referred to the local hospital department regarding upper arch crowding. Plain film radiographs confirmed the congenital absence of both lower lateral incisors in addition to an unerupted conical supernumerary tooth in the maxillary midline. This condition has been called hypo-hyperdontia and in this paper, we discuss the clinical findings and treatment planning considerations in relation to the limited number of previously reported cases. The case report raises awareness of concomitant hypo-hyperdontia and serves to highlight that concomitant anomalies should be excluded when hypodontia or supernumerary teeth are diagnosed.

## 1. Introduction

Hypodontia and supernumerary teeth are often considered as two extremes of the same type of dental anomaly. They most commonly occur as mutually exclusive conditions, and each represents its own challenge in terms of treatment planning. On very rare occasions, hypodontia and supernumerary teeth can coexist with the condition termed “concomitant hypodontia and hyperdontia” [1], “oligopleiodontia” [2], or “hypo-hyperdontia” [3]. More recently, the term concomitant hypo-hyperdontia (CHH) has been used. Being an anomaly of extremes, CHH has been associated with conditions such as Ellis-van Creveld syndrome [4], Marfan syndrome [5], and Downs syndrome [6]. Nonsyndromic CHH is very rare with one epidemiological study finding an incidence of 0.3% in an orthodontic population [7]. It is therefore likely that the true incidence in the general population is significantly lower. The aim of this case report is to present a rare finding of CHH in a nonsyndromic adolescent who was referred as part of a routine orthodontic referral. The clinical presentation in relation to previously reported cases is discussed to raise awareness of the condition.

## 2. Case Report

An 11-year-old, white male was referred to the Department of Child Dental Health, at the local University Dental Hospital for an orthodontic opinion from his general dental practitioner. The medical, dental, and social histories were unremarkable.

### 2.1. Extraoral Assessment

The patient presented with a class I skeletal relationship with an average Frankfort-mandibular planes angle and lower facial height proportion. There was no significant asymmetry in the transverse plane. The lips were competent, and the nasolabial angle was slightly reduced. An assessment of the temporomandibular joint was unremarkable.

### 2.2. Intraoral Assessment

**Figure 1:**
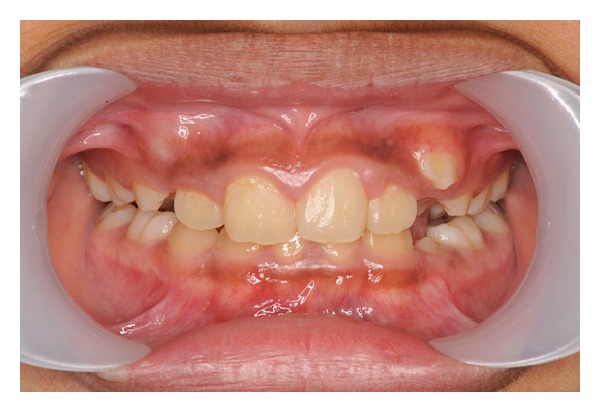
Anterior occlusal photograph of dentition.

In occlusion the patient had a mild class II division 2 incisor relationship in the mixed dentition, with an overjet of 2 mm and an increased, complete to tooth overbite (Figure 1). The upper dental centreline was coincident with the facial midline while the lower dental centreline was displaced 3 mm to the left of the upper.

**Figure 2:**
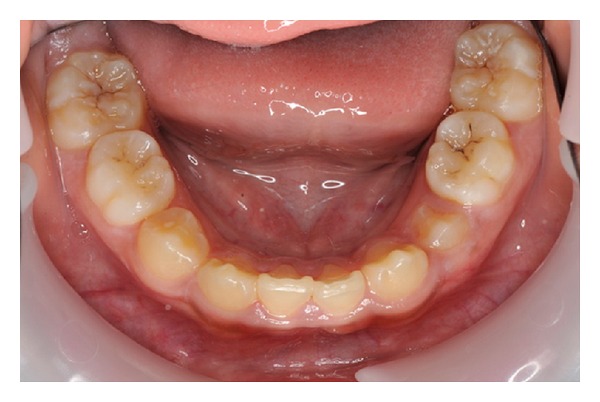
Lower occlusal photograph of dentition showing clinical absent lower lateral incisors.

The lower arch was essentially well aligned; however, both lower permanent lateral incisors were clinically absent (Figure 2).

**Figure 3:**
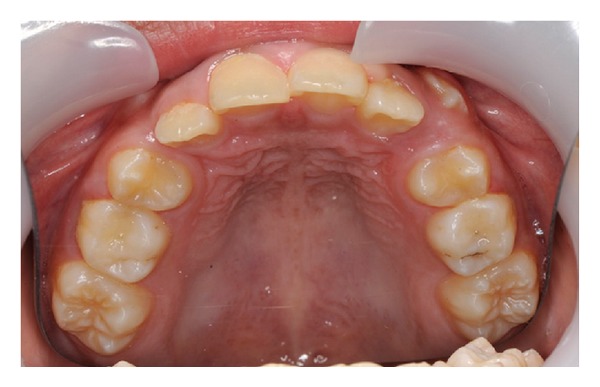
Upper occlusal photograph showing crowded permanent canines.

The upper arch was severely crowded with a lack of space for both upper permanent canines (Figure 3). 

The patient's oral hygiene was fair with small amounts of plaque being present in the cervical margins of the posterior dentition. There was occlusal fissure caries present in the lower first permanent molars.

### 2.3. Radiographic Assessment

**Figure 4:**
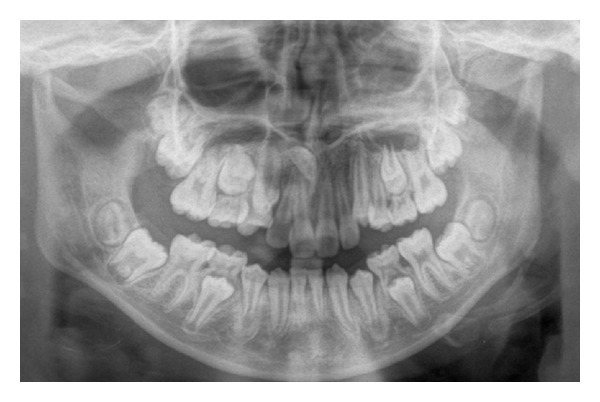
Dental pantomogram confirming congenitally absence of lower lateral incisors and upper midline supernumerary.

A dental pantomogram (Figure 4) revealed the presence of all permanent teeth excluding LL2 and LR2. In addition, an inverted conical supernumerary was found in the maxillary midline close to the root apices of the UR1 and UR2. There was no obvious root resorption of the UR1 and UR2, and all other root lengths appeared normal for the patient's chronological age. Molar stacking was evident in both upper quadrants.

### 2.4. Treatment Plan

The clinical and radiographic findings were explained to the patient and parents. Oral hygiene instruction, diet advice, and tooth brushing instruction were given. Bite-wing radiographs followed by preventive resin restorations were planned for all four first permanent molars.

Given the reasonable lower arch alignment and aesthetics despite congenitally absent lower lateral incisors, the lower arch was to be treated on a nonextraction basis. Removal of the maxillary first premolars was requested to allow spontaneous eruption of the maxillary permanent canines. There was a discussion with regard to the proximity of the upper midline supernumerary and the roots of the upper incisors. If fixed appliance therapy was to be considered, then the supernumerary would most likely require removal. As the patient and parents were apprehensive regarding a general anaesthetic, the decision was made to review the interarch relationships in a 6-month time and confirm active treatment or accept the malocclusion with regular monitoring of the upper incisor roots and supernumerary.

## 3. Discussion

The authors have presented a case of CHH in a nonsyndromic patient. The incidence of such a condition in the general population is unknown but is likely to be very rare given the incidence in an orthodontic population has been documented as low as 0.3% [7]. The aetiology of CHH is unclear underlying the complex mechanisms causing hypodontia and supernumerary teeth. The aetiological factors associated with hypodontia include physical disruption of the lamina dura, abnormalities of the dental epithelium, and underlying neural crest derived mesenchyme failing to induce tooth germ production [20]. There is a strong genetic basis with *MSX1*, *TGFA*, and *PAX9 *mutations isolated for tooth agenesis [21]. More recently, WNT10A has been isolated and shown to improve the diagnostic yield of DNA testing in isolated nonsyndromic hypodontia [22]. The aetiology of supernumerary teeth includes the phylogenetic theory, the dichotomy theory, and the presence of an overactive lamina dura [23]. Despite a strong genetic basis behind both hypodontia and supernumerary teeth, a study looking at nine patients with CHH found that none of the family members also suffered from CHH [24]. From the current limited literature, CHH seems to be a result of disturbances to neural crest migration, proliferation, and differentiation when tooth germ formation is initiated [17]. In addition, there is evidence to suggest a model of tooth size and shape discrepancies between hypodontia and supernumerary patients. The more severe the hypodontia, the smaller the mesiodistal width of the teeth formed. Conversely, patients with supernumerary teeth tend to have significantly larger maxillary central and lateral incisors than controls [25].

Whilst hypodontia is more common amongst females and supernumerary amongst males, CHH has been reported to affect both genders equally [25].

Table 1 summarises the phenotypic presentation of nonsyndromic cases of CHH reported in the literature over the last 20 years. Two cases of CHH have been reported in the primary dentition, although this is believed to be extremely rare [15]. Abnormalities of the primary dentition are almost certainly likely to be translated to the permanent dentition. The majority of other cases of CHH have presented with missing upper lateral incisors and/or missing lower second premolars. This correlates with teeth most commonly absent in isolated hypodontia [20]. In a similar manner, the most common supernumerary present in CHH appears to be a mesiodens, which mirrors the common presentation of an isolated supernumerary tooth [23]. 

One case of CHH has reported that a mesiodens was found in conjunction with a missing maxillary lateral incisor [11]. The aetiology of this occurrence of CHH has been speculated as a transposition. However, as the majority of reported cases of CHH are in opposing arches, this theory is unlikely. 

Fortunately in the case presented here, the diagnosis of CHH does not seem to have impacted significantly on the treatment plan. Although the supernumerary present was a classic feature of an additional tooth to the normal series, the pattern of hypodontia was not typical. Isolated incidences of congenitally absent mandibular incisors are believed to be in the region of 0.08–0.23% [20], and therefore the presentation is likely to be quite rare. 

The body of knowledge on CHH is steadily growing. Literature reviews and case series have helped to recognise a condition which was once thought of as two opposite entities. 

## 4. Conclusion

This case report highlights a routine orthodontic referral presenting with simultaneous occurrence of hypodontia and a supernumerary tooth. Practitioners should be aware that hypodontia and supernumerary teeth may not be mutually exclusive in their presentation.

## Figures and Tables

**Table 1:** Presentation of nonsyndromic CHH in published case reports in English language literature over the last 20 years.

Author	Year	Gender	Age	Hypodontia		Supernumeraries	
				Maxilla	Mandible	Maxilla	Mandible
Hewson et al. [8]	1995	Male	9		LR5	Central incisor	
Zhu et al. [9]	1996	Male	14	UR2, UL2			UL6 region
Scheiner and Sampson [10]	1997	Female	8		LL5, LR5	2 mesiodens	
Segura and Jiménez-Rubio [11]	1998	Male	13	UL2		Mesiodens	
Matsumoto et al. [12]	2001	Female	8	UL5	LL2	UL2	
Sharma [13]	2001	Female	12	UL3		Six supernumeraries	
Oliveira et al. [14]	2002	Female	9		LL5, LR5	Mesiodens	
	2002	Male	8	Gemination		2 mesiodens	
El-Bahannasawy and Fung [15]	2004	Female	4	URC		URD, UR5	
Patchett et al. [16]	2006	Male	9		LL5, LR5	Mesiodens	
Anthonappa et al. [17]	2008	Female	12		LL1, LR1	UL3	
		Male	9	UR5	LL5	UL1	
		Male	11		LL2	UR5	
		Male	5		LLA, LRA, LR2	Mesiodens	
		Male	7		LL1, LR1	2 mesiodens	
		Female	5		LLB, LL2	Mesiodens	
		Male	6		LL5, LR5	Mesiodens	
Varela et al. [7]	2009	Female	Not given		LL5	LR2	
		Male	Not given		LL5, LR5	Mesiodens	
		Male	Not given		LL5	UR2	
		Male	Not given		LL5, LR5		LL2
		Male	Not given	UL2		Mesiodens	
		Female	Not given	UL2		ULB	
		Female	Not given	UL2		URB, UR2	
Marya et al. [18]	2012	Male	20		LL1, LR1		Mesiodens
Nirmala et al. [19]	2013	Female	13	UR3, UL3		Mesiodens	

LL: lower left, LR: lower right, UL: upper left, UR: upper right.
